# Impfbereitschaft von Krankenhauspersonal in Deutschland: Welche Rolle spielen Verschwörungsannahmen zu COVID-19?

**DOI:** 10.1007/s00103-022-03593-0

**Published:** 2022-10-07

**Authors:** Julia Petersen, Lina Marie Mülder, Peter Kegel, Nikolaus Röthke, Hauke Felix Wiegand, Klaus Lieb, Henrik Walter, Anna-Lena Bröcker, Susanne Liebe, Oliver Tüscher, Andrea Pfennig, Birgit Maicher, Sabine Hellwig, Frank Padberg, Kristina Adorjan, Stefan Unterecker, Paula Wessels, Dirk-Matthias Rose, Manfred E. Beutel

**Affiliations:** 1 Klinik und Poliklinik für Psychosomatische Medizin und Psychotherapie, Universitätsmedizin der Johannes Gutenberg-Universität Mainz, Untere Zahlbacher Straße 8, 55131 Mainz, Deutschland; 2 Abteilung für Arbeits‑, Organisations- und Wirtschaftspsychologie, Institut für Psychologie, Johannes Gutenberg-Universität Mainz, Mainz, Deutschland; 3 Institut für Arbeits‑, Sozial- und Umweltmedizin, Universitätsmedizin, Johannes Gutenberg-Universität Mainz, Mainz, Deutschland; 4 Klinik für Psychiatrie und Psychotherapie, Universitätsmedizin, Johannes Gutenberg-Universität Mainz, Mainz, Deutschland; 5 Klinik für Psychiatrie und Psychotheraphie, CCM, Charité-Universitätsmedizin Berlin, corporate member of Freie Universität Berlin and Humboldt-Universität zu Berlin, Berlin, Deutschland; 6 Universitätsklinikum Carl Gustav Carus, Technische Universität Dresden, Dresden, Deutschland; 7 Klinik und Poliklinik für Psychiatrie und Psychotherapie, Universitätsklinikum Carl Gustav Carus, Technische Universität Dresden, Dresden, Deutschland; 8 Klinik für Psychiatrie und Psychotherapie, Universitätsklinikum Freiburg, Medizinische Fakultät, Albert-Ludwigs-Universität Freiburg, Freiburg, Deutschland; 9 Klinik für Psychiatrie und Psychotherapie, Ludwig-Maximilians-Universitätsklinikum München, München, Deutschland; 10 Klinik und Poliklinik für Psychiatrie, Psychosomatik und Psychotherapie, Universitätsklinikum Würzburg, Würzburg, Deutschland

**Keywords:** COVID-19-Impfung, Gesundheitspersonal, Prädiktoren, Verschwörungsannahmen, Kommunikation, COVID-19 vaccination, Healthcare workers, Predictors, Conspiracy theories, Communication

## Abstract

**Hintergrund:**

Zur Erreichung einer flächendeckenden Immunität gegen COVID-19 in der Bevölkerung ist entscheidend, wie sich die Impfbereitschaft bislang Ungeimpfter entwickelt. Schlüsselrolle dabei spielt das medizinische Personal, welches die Gesundheitsversorgung während der Pandemie gewährleistet und vielen Menschen als Informationsquelle zu Impfungen dient. Die allgemeine Impfbereitschaft wird u. a. negativ beeinflusst durch Verschwörungsannahmen und die Verbreitung von Desinformationen.

**Ziele:**

Es wurden Impfbereitschaft und verschiedene Einflussgrößen bei Klinikpersonal in Deutschland untersucht, um Hinweise auf eine mögliche Steigerung der Impfbereitschaft zu erlangen.

**Methoden:**

Im Rahmen des vom Bundesministerium für Bildung und Forschung geförderten egePan-Verbundprojekts des nationalen Netzwerkes Universitätsmedizin wurden zwischen Januar und Juni 2021 in einer freiwilligen, anonymen Onlinebefragung die Impfbereitschaft, individuelle Sozialmerkmale, Zustimmung zu Verschwörungsannahmen und Fragen zur Kommunikation in deutschen Kliniken erhoben.

**Ergebnisse:**

Insbesondere Ärzt*innen und wissenschaftliches Personal gaben eine erhöhte Impfbereitschaft im Vergleich zur Gesamtbevölkerung an. Verschwörungsannahmen waren kaum verbreitet, am häufigsten jedoch unter dem Verwaltungs- und Pflegepersonal. Verschwörungsannahmen waren negativ assoziiert mit der Impfbereitschaft. Prädiktoren für eine höhere Impfbereitschaft waren die empfundene Sicherheit und Effektivität von Impfungen sowie ein höheres Alter.

**Diskussion:**

Da sich empfundene Sicherheit und Effektivität von Impfungen positiv auf die Impfbereitschaft auswirkten, könnten eine dahingehende Aufklärungsarbeit und transparente Informationsvermittlung der Verbreitung von Verschwörungsannahmen entgegenwirken und die Impfraten unter Krankenhauspersonal erhöhen.

## Abstract (en)

**Background:**

A critical factor in achieving widespread immunity against COVID-19 is the willingness of previously unvaccinated individuals to get vaccinated. Medical staff play a key role in this, as they ensure healthcare during the pandemic and for many serve as a source of information about vaccinations. Among the factors that negatively influence the general willingness to get vaccinated are conspiracy assumptions and the spread of misinformation.

**Objective:**

The willingness of hospital staff in Germany to get vaccinated and various influencing variables were examined to obtain indicators that could help increase the general willingness to get vaccinated.

**Methods:**

Between January and June 2021, a voluntary and anonymous online survey conducted as part of the egePan joint project of the national network for university medicine (funded by the Federal Ministry of Education and Research) was used to assess the willingness to be vaccinated, individual social characteristics, the belief in conspiracy assumptions, and communication items in German hospitals.

**Results:**

In comparison with the general population, physicians and scientific staff in particular indicated an increased willingness to get vaccinated. Conspiracy assumptions were not very widespread but most frequent among administrative and nursing staff. Conspiracy assumptions were negatively associated with the willingness to get vaccinated. Predictors for a higher willingness to get vaccinated were the perceived safety and effectiveness of vaccinations and a higher age.

**Discussion:**

Since the perceived safety and effectiveness of vaccinations have a positive effect on the willingness to get vaccinated, educational work and transparent information transfer could counteract the spread of conspiracy assumptions and increase vaccination rates among hospital staff.

## Hintergrund

Um die COVID-19-Pandemie bewältigen zu können, muss eine flächendeckende Immunität der Bevölkerung („Herdenimmunität“), beispielsweise durch Impfungen, erreicht werden [1, 2]. Dennoch wollen sich eine relevante Anzahl von Personen nicht impfen lassen und äußern teilweise ihre Ablehnung auch in öffentlichen Protesten [3]. Zur Steigerung der Impfbereitschaft ist es daher notwendig, die generelle Entwicklung der Impfbereitschaft sowie Gründe der Ablehnung zu verstehen [1, 2, 4]. Eine europäische Befragung zeigte, dass in Deutschland und Frankreich anteilig die meisten Impfverweiger*innen zu verzeichnen sind [2]. Eine positive Einstellung zum Impfen war assoziiert mit bestimmten soziodemografischen Faktoren, dem Wissen über die Impfung und Verschwörungsannahmen sowie dem Vertrauen in öffentliche Institutionen und Informationen.

Nach der Theorie der Schutzmotivation (*Protection Motivation Theory*, PMT) sind Personen besonders dann zu Schutzmaßnahmen wie dem Impfen bereit, wenn sie überzeugt sind, dass sich nicht impfen zu lassen mit negativen Konsequenzen oder Gefahren einhergeht, die durch das Impfen reduziert werden [5]. Die Impfintention wird dabei beeinflusst vom eigenen Wissen über Impfungen und Risiken sowie der Einschätzung der Bedrohung durch das Virus, der empfundenen Impf- und der Selbstwirksamkeit [5, 6]. In einer Studie begründeten Personen ihre Unsicherheit gegenüber der Impfung mit der Sorge über mögliche Nebenwirkungen, Sicherheit und Effektivität der Impfstoffe [2]. 11 % waren zudem der Ansicht, dass COVID-19 für sie nicht gesundheitsgefährdend sei [2]. Impfwillige waren tendenziell männlich und älter, während jüngere und weibliche Befragte sich impfunsicher zeigten [2, 7]. Weitere Studien verwiesen auf den Zusammenhang einer höheren Impfbereitschaft mit einem höheren Bildungsabschluss, der Zugehörigkeit zu einer Risikogruppe für einen schweren COVID-19-Verlauf sowie Infektionen bei sich selbst oder nahestehenden Angehörigen [8, 9].

Insbesondere die Impfbereitschaft des medizinischen Personals spielt eine Schlüsselrolle für die Bewältigung der COVID-19-Pandemie: Nicht nur gewährleisten sie die medizinische Versorgung während der Pandemie und stehen damit unter einem besonderen Infektionsrisiko, sie sind zudem erste Ansprechpartner*innen für viele Bürger*innen, die Informationen über die Impfungen einholen wollen [10]. Bezüglich der Impfbereitschaft des medizinischen Personals lassen sich ähnliche Ergebnisse wie in der Gesamtbevölkerung beobachten, die Impfbereitschaft innerhalb dieser Gruppe ist jedoch insgesamt höher. Im Vergleich zum ärztlichen Personal ist das nichtärztliche Krankenhauspersonal zurückhaltender gegenüber der Impfung [11–13]. Eine erhöhte Impfbereitschaft lag gemäß der Theorie der Schutzmotivation vor, wenn der soziale Vorteil des Impfens als hoch und die Aktionskosten des Impfens (wie beispielsweise Aufwand und Risiken) als gering eingeschätzt wurden [14, 15]. Umgekehrt waren hohe Aktionskosten im Vergleich zum gewonnenen Schutz durch die Impfung negativ mit der Impfbereitschaft assoziiert [14, 16]. Diese Ergebnisse werden durch Studien unterstützt, die einen Zusammenhang zwischen fehlender Impfbereitschaft und der Sorge über mögliche Nebenwirkungen, wie beispielweise Allergien, aufwiesen [17]. Eine während der deutschen COVID-19-Impfkampagne im Frühjahr 2021 besonders publik gewordene Nebenwirkung ist die Hirnvenenthrombose, die laut Paul-Ehrlich-Institut (PEI) im Zusammenhang mit dem Vektorimpfstoff Vaxzevria (ehemals COVID-19 Vaccine AstraZeneca^1^) vor allem bei jüngeren Frauen auftrat [18]. Daher wurden Impfungen mit diesem Impfstoff in Deutschland am 14.03.2021 zunächst sicherheitshalber ausgesetzt und ab April 2021 – nach ausgiebiger Prüfung durch die Europäische Arzneimittel-Agentur (EMA) und die Ständige Impfkommission (STIKO) – eingeschränkt für Menschen über 60 Jahre wieder empfohlen. Eine AstraZeneca-Impfung von Personen auch unterhalb dieser Altersgrenze bleibt nach ärztlicher Aufklärung und bei individueller Risikoakzeptanz möglich [18].

Diese Entwicklungen hatten jedoch einen negativen Einfluss auf die Impfbereitschaft: Teilweise fühlten sich Personen verunsichert und misstrauten den Empfehlungen bezüglich des AstraZeneca-Impfstoffes [19]. Die Impfbereitschaft wurde ebenfalls negativ beeinflusst durch die Verbreitung von Desinformationen [12], die zum Teil auf Verschwörungsannahmen zurückgehen [1]. Verschwörungsannahmen verbreiten sich vor allem in Krisensituationen [20, 21] und beziehen sich auf den Glauben, dass eine Gruppe mächtiger Akteur*innen mit böswilligen Absichten hinter aktuellen und/oder historischen Ereignissen steht [22]. Vor allem Personen, die sich hilflos fühlen, glauben an derartige Theorien in dem Versuch, eine komplexe, unkontrollierbare Krisensituation nachvollziehen zu können [22].

Die COVID-19-Pandemie ist insofern eine Krisensituation, da sie neuartige, unkontrollierbare Herausforderungen an das öffentliche Gesundheitssystem stellte und zum Teil kontroverse politische Entscheidungen zu Eindämmungsmaßnahmen nach sich zog, welche in individuelle Grundfreiheiten eingriffen (wie beispielsweise Abstandsgebote und Einschränkungen der Versammlungsfreiheit). Dies traf auf eine zunehmend verbreitete Bewegung, welche Mede und Schäfer (2020) als Wissenschaftspopulismus bezeichnen [23]. Laut dem Wissenschaftspopulismus gibt es einen fundamentalen Konflikt zwischen „normalen“ Bürger*innen, die ihre Entscheidungen auf Basis von vermeintlichem Konsens und alltäglichen Erfahrungen treffen, und der unmoralischen akademischen Elite, welche „unbrauchbares und unauthentisches wissenschaftliches Wissen“ [18] verbreiten und sich mit anderen Mitgliedern der Elite verbünden [24]. Umstritten in diesem Konflikt ist zudem die Macht darüber, entscheiden zu können, was als „wahres“ Wissen angesehen wird, da die akademischen Eliten angeblich lediglich ideologiebasiertes Wissen produzieren und der Bevölkerung viele Fakten vorenthalten [23, 24].

Das entstehende Gefühl, die Kontrolle über eine Situation zu verlieren, ist erwiesenermaßen mit dem Glauben an Verschwörungstheorien assoziiert [22, 23]. Dies kann im Kontext der COVID-19-Pandemie beispielsweise dazu führen, dass Wissenschaftler*innen als Konspirateur*innen angesehen werden und stattdessen an Desinformationen geglaubt wird. Vorangegangene Studien zeigten, dass COVID-19-bezogene Verschwörungsannahmen unter anderem den Standpunkt vertreten, dass die Tragweite der Pandemie und die Gefahr von SARS-CoV‑2 weitaus geringer seien als in der Öffentlichkeit dargestellt und sich einige wenige Personen an der Krise bereichern würden [1, 25]. In einer aktuellen Bevölkerungsstudie teilten beispielsweise 7 % der Befragten Verschwörungsannahmen wie, „[die] Corona-Krise wurde so groß geredet, damit einige wenige von ihr profitieren“ bzw. „[die] tatsächlichen Hintergründe der Corona-Erkrankung werden nie ans Licht der Öffentlichkeit kommen“ (35,8 %; [1]). Menschen, die solchen Aussagen zustimmten, wiesen auch eine geringere Impfbereitschaft auf [1].

Aufgrund ihrer medizinischen Sachkenntnis und der Konfrontation mit schweren und tödlichen COVID-19-Verläufen ist anzunehmen, dass Beschäftigte im Gesundheitssystem weniger empfänglich für Verschwörungsannahmen sind als medizinische Laien. Umso wichtiger ist es zu prüfen, welche Faktoren die Impfbereitschaft unter medizinischem Personal beeinträchtigen und ob dies ähnliche Faktoren wie in der Bevölkerung sind bzw. welche Rolle die Kommunikation innerhalb der Kliniken als institutioneller Faktor spielt.

Die vorliegende Arbeit untersucht den Zusammenhang der Impfbereitschaft von Krankenhauspersonal mit soziodemografischen und beruflichen Faktoren, dem Glauben an Verschwörungsannahmen, Überzeugungen von Sicherheit und Wirksamkeit der Impfung sowie dem Vertrauen in die Kommunikation innerhalb der Klinik. Dafür stellen wir auf Basis der oben zitierten Literatur folgende Hypothesen auf:

1. Impfbereite Menschen sind häufiger männlich und älter. Ärztliches Personal hat im Vergleich zu anderem Krankenhauspersonal die höchste Impfbereitschaft.
2. Je wirksamer und sicherer ein Impfstoff wahrgenommen wird, desto höher ist die Impfbereitschaft.
3. Die Impfbereitschaft ist negativ mit Verschwörungsglauben assoziiert.
4. Eine gute Kommunikation innerhalb der Kliniken steigert das Vertrauen, was wiederum mit einer hohen Impfbereitschaft einhergeht.
5. Der Zeitpunkt der Befragung spielt eine Rolle: Wir gehen davon aus, dass die Impfbereitschaft nach dem sicherheitsbedingten Aussetzen der Impfungen mit dem AstraZeneca-Impfstoff gesunken ist.

## Methoden

### Studiendesign

Im Rahmen des vom Bundesministerium für Bildung und Forschung (BMBF) geförderten egePan-Verbundprojekts *(Entwicklung, Testung und Implementierung von regional adaptiven Versorgungsstrukturen und Prozessen für ein evidenzgeleitetes Pandemiemanagement koordiniert durch die Universitätsmedizin)* des nationalen Netzwerkes Universitätsmedizin (NUM) wurde zwischen Januar und Juni 2021 eine Onlinebefragung von Mitarbeiter*innen im Gesundheitswesen durchgeführt. Hierfür wurden die egePan-Netzwerkpartner*innen darum gebeten, die Studie in ihrer Klinik bekannt zu machen und den Link zur Umfrage zu versenden. Die Befragung erfolgte auf freiwilliger Basis und anonym. Es nahmen insgesamt 2011 Krankenhausmitarbeiter*innen an der Umfrage teil. Der *Missing-Completely-At-Random*-(MCAR-)Test [26] zeigte, dass die fehlenden Werte für die Impfbereitschafts‑, Verschwörungsannahme- und Kommunikationsitems vollkommen zufällig fehlten. Deswegen wurde ein fallweiser Ausschluss (*Listwise Deletion*) vorgenommen, welcher uns auf 1683 einbezogene Fälle brachte.

### Messinstrumente

Die *Impfbereitschaft* des Krankenhauspersonals wurde erhoben auf Basis des an die COVID-19-Snapshot-Monitoring-(COSMO-)Studie angelehnten Items [27]: „Wie würden Sie entscheiden, wenn Sie nächste Woche die Möglichkeit hätten, sich mit [einem] Impfstoff gegen das Coronavirus impfen zu lassen?“ Ergänzend wurde in dieser Studie nach der empfundenen Sicherheit der Impfung („Ich habe vollstes Vertrauen, dass eine Impfung gegen COVID-19 sicher ist“) sowie der empfundenen Effektivität der Impfung („Ich bin davon überzeugt, dass die Impfung gegen COVID-19 effektiv ist“) gefragt. Die Einschätzung erfolgte jeweils auf einer 7‑stufigen Likert-Skala (1 = *stimme überhaupt nicht zu bzw. auf gar keinen Fall;* 7 = *stimme voll und ganz zu bzw. auf jeden Fall*). Das erste Item wurde in der Regressionsanalyse als Outcome-Variable verwendet, die Sicherheits- und Effektivitäts-Items als erklärende Variablen gemäß der Theorie der Schutzmotivation als Faktoren der Selbstwirksamkeit- und Handlungsergebniserwartung.

Die Fragen zum *Glauben an Verschwörungsannahmen* wurden unter anderem als Faktoren der Bedrohungseinschätzung ebenfalls dem Fragebogen der COSMO-Studie entnommen. Die Zustimmung zu den Items: „Das Virus wird absichtlich als gefährlich dargestellt, um die Öffentlichkeit in die Irre zu führen“, „Expert*innen täuschen uns absichtlich und zu ihrem eigenen Vorteil, obwohl das Virus eigentlich nicht schlimmer ist als eine Grippe“, und „Man sollte den Expert*innen glauben, wenn sie sagen, dass das Virus gefährlich ist“, wurde ebenfalls mittels einer 7‑stufigen Likert-Skala erhoben (von 1 = *stimme überhaupt nicht zu;* 7 = *stimme voll und ganz zu*; [27]). Ergänzt wurde dies mit der Aussage: „Die Corona-Krise wurde so groß geredet, damit einige wenige von ihr profitieren“ ([1, 25]; gleiche Skalierung). Die Items wurden für die Regressionsanalyse zu einem aggregierten Index zusammengefasst (Cronbach’sches Alpha = 0,88).

Die *Kommunikation innerhalb der Klinik* wurde mit 6 Aussagen erhoben: „Ich fühle mich ausreichend über die Maßnahmen im Krankenhaus informiert“, „Entscheidungsabläufe und Informationen sind für mich transparent“, „Ich werde immer rechtzeitig über Entwicklungen/Entscheidungen informiert“, „Ich kann meine eigenen Erfahrungen und Ideen im Zusammenhang mit COVID-19 einbringen“, „Der Informationsfluss von Führungskräften zu Mitarbeitenden funktioniert gut“ sowie „Der Informationsfluss von Geschäftsführung/Vorstand zu Mitarbeitenden funktioniert gut“. Auch hier wurde eine 7‑stufige Likert-Skala (1 = *stimme überhaupt nicht zu*; 7 = *stimme voll und ganz zu*) verwendet. Die Items wurden für die Regressionsanalyse zu einem aggregierten Index zusammengefasst (Cronbach’sches Alpha = 0,83).

Zur Bestimmung des zeitlichen Verlaufs der Impfbereitschaft wurde der Zeitraum vor und nach dem vorsorglichen Aussetzen der Impfungen mit AstraZeneca für Personen unter 60 Jahren in Deutschland (14.03.2021) unterschieden [1, 18]. Hierfür wurde die Stichprobe in 2 annähernd gleich große Teile geteilt.

### Datenanalyse

Mithilfe der Statistiksoftware R (Version 1.3.1056) wurde zunächst die Deskriptivstatistik für die relevanten Variablen berechnet. Hierbei wurden zudem die Mittelwerte (M) und Standardabweichungen (SD) für die Impfeinstellungs‑, Verschwörungs-, und Kommunikations-Items berechnet sowie Mittelwertvergleiche durchgeführt. Die Signifikanz (*p*) der Unterschiede wurde dabei mittels t‑Tests und Varianzanalyse (ANOVA) berechnet. Zusätzlich wurden für alle Faktoren noch die Effektstärken Cohens d [28] und Eta-Quadrat (η^2^) kalkuliert. Daraufhin wurden Assoziationen zwischen Items mittels Korrelationen (Spearman Rangkorrelationskoeffizient rho) überprüft.

Als letzter Analyseschritt wurde eine multiple lineare Regressionsgleichung mit dem Impfbereitschafts-Item als Outcome-Variable und dem aggregierten Verschwörungs- und dem aggregierten Kommunikations-Index sowie den einzelnen Impfeinstellungs-Items, der Zeitvariablen und den soziodemografischen Variablen als erklärende Variablen durchgeführt. Zuvor wurde auf Normalverteilung, Multikollinearität und Homoskedastizität getestet. Es wurde dabei lediglich eine Heteroskedastizität nachgewiesen, weshalb für die Regression die robusten Standardfehler ausgegeben wurden, um einem Fehler 2. Art entgegenzuwirken. Im Anschluss an die Regressionsanalyse wurden zudem die Koeffizienten zur Vergleichbarkeit standardisiert.

## Ergebnisse

### Stichprobe

Die Stichprobe umfasste zu einem größeren Anteil Frauen (78,7 %). Erwartungsgemäß waren über 60-Jährige unterrepräsentiert, da die Mehrzahl der Berentungen Anfang bis Mitte 60 erfolgen [29]. Bezüglich Alter und Geschlecht unterscheidet sich die Stichprobe jedoch nicht vom Personal stationärer und teilstationärer Einrichtungen in Deutschland nach Stand 2019 [30], womit die Stichprobe als demografisch vergleichbar angesehen werden kann. Die Untersuchungspopulation umfasste Pflege- (32,8 %), Verwaltungs- (24,2 %) und medizinisch-technisches Personal (19,0 %), Ärzt*innen (13,4 %) sowie wissenschaftliches Personal (10,6 %). 63,0 % der Befragten gaben an, Kontakt zu COVID-19-Patient*innen zu haben oder gehabt zu haben. 6,9 % hatten zum Befragungszeitpunkt selbst eine SARS-CoV-2-Infektion durchgemacht. Insgesamt war die Gesamtstichprobe durch eine relativ hohe Impfbereitschaft geprägt (M = 6,29, SD = 1,59). Die Mittelwerte für Impfbereitschaft, Annahmen zu Effektivität und Sicherheit der Impfung sowie für die Verschwörungs-Items sind in Tab. 1 dargestellt.

**Table 1:** 

	Anteil *n* (%)	Impfbereitschaft M (SD)	Sicherheit M (SD)	Effektivität M (SD)	Als gefährlicher dargestellt M (SD)	Expert*innen täuschen M (SD)	Expert*innen glauben M (SD)	Corona-Profit M (SD)
**Gesamtstichprobe (*N*** **=** **1683)**	–	6,29 (1,59)	5,60 (1,58)	5,44 (1,69)	1,68 (1,36)	1,57 (1,27)	2,05 (1,61)	2,00 (1,55)
*Geschlecht*	–	*d*_*Cohen*_ *=* *0,208*	*d*_*Cohen*_ *=* *0,210*	*d*_*Cohen*_ *=* *0,250*	*d*_*Cohen*_ *=* *−0,124*	*d*_*Cohen*_ *=* *−0,142*	*d*_*Cohen*_ *=* *−0,037*	*d*_*Cohen*_ *=* *−0,187*
Weiblich	1324 (78,7 %)	6,22 (1,67)***	5,53 (1,62)***	5,35 (1,72)***	1,72 (1,40)***	1,61 (1,31)***	2,06 (1,62)***	2,06 (1,58)***
Männlich	359 (21,3 %)	6,55 (1,23)***	5,86 (1,37)***	5,77 (1,51)***	1,55 (1,23)***	1,43 (1,09)***	2,00 (1,56)***	1,77 (1,42)***
*Altersgruppen (Jahre)*	*–*	η^2^ = 0,009	η^2^ = 0,002	η^2^ = 0,002	η^2^ = 0,003	η^2^ = 0,002	η^2^ = 0,000	η^2^ = 0,000
18–30	309 (18,4 %)	6,06 (1,77)*^a,b^	5,49 (1,65)	5,33 (1,77)	1,84 (1,48)	1,72 (1,42)	2,06 (1,52)	2,04 (1,53)
31–40	452 (26,9 %)	6,20 (1,75)	5,58 (1,54)	5,38 (1,68)	1,72 (1,35)	1,58 (1,22)	2,07 (1,54)	1,99 (1,51)
41–50	381 (22,6 %)	6,33 (1,51)	5,67 (1,58)	5,53 (1,69)	1,61 (1,29)	1,50 (1,20)	2,09 (1,62)	2,01 (1,54)
51–60	429 (15,5 %)	6,44 (1,41)	5,61 (1,60)	5,45 (1,57)	1,61 (1,30)	1,53 (1,22)	2,02 (1,70)	1,97 (1,57)
61–70	112 (6,6 %)	6,56 (1,24)	5,79 (1,37)	5,61 (1,51)	1,62 (1,55)	1,56 (1,41)	1,88 (1,72)	2,00 (1,80)
*Berufsgruppe*	–	η^2^ = 0,005	η^2^ = 0,003	η^2^ = 0,002	η^2^ = 0,006	η^2^ = 0,005	η^2^ = 0,001	η^2^ = 0,008
Ärzt*innen	226 (13,4 %)	6,81 (0,78)***^cde^	6,15 (1,13)	5,99 (1,22)***^cde^	1,32 (0,94)*^c,^***^de^	1,22 (0,80)*^c,^***^de^	1,72 (1,38) *^d,^***^e^	1,40 (0,94)***^cde^
Pflegepersonal	552 (32,8 %)	6,22 (1,65)	5,49 (1,65)	5,32 (1,76)*^f^	1,63 (1,29)*^e^	1,52 (1,19)*^d,^**^e^	2,05 (1,60)	1,97 (1,49)**^e,^*^f^
Medizinisch-technisches Personal	319 (19,0 %)	6,26 (1,66)	5,52 (1,65)	5,34 (1,74)	1,86 (1,44)*^f^	1,77 (1,41)**^f^	2,16 (1,60)*^f^	2,24 (1,69)***^f^
Verwaltung	407 (24,2 %)	6,05 (1,85)*^f^	5,39 (1,64)	5,22 (1,76)*^f^	1,90 (1,59)*^f^	1,79 (1,47)***^f^	2,29 (1,81)***^f^	2,35 (1,80)***^f^
Wissenschaftliches Personal	179 (10,6 %)	6,46 (1,34)	5,90 (1,35)	5,75 (1,53)	1,51 (1,21)	1,32 (1,04)	1,69 (1,22)	1,60 (1,18)
Kontakt zu COVID-19-Patient*innen (N *=* *1114)*	–	*d*_*Cohen*_ *=* *−0,095*	*d*_*Cohen*_ *=* *−0,031*	*d*_*Cohen*_ *=* *−0,123*	*d*_*Cohen*_ *=* *0,054*	*d*_*Cohen*_ *=* *0,016*	*d*_*Cohen*_ *=* *0,032*	*d*_*Cohen*_ *=* *0,052*
Ja	702 (63,0 %)	6,37 (1,53)	5,65 (1,60)	5,53 (1,69)*	1,62 (1,30)	1,55 (1,26)	2,01 (1,57)	1,95 (1,52)
Nein	412 (37,0 %)	6,22 (1,67)	5,60 (1,58)	5,32 (1,73)*	1,69 (1,32)	1,57 (1,22)	2,06 (1,59)	2,03 (1,54)
*Positiver COVID-19-Test (N* *=* *1190)*	–	*d*_*Cohen*_ *=* *0,261*	*d*_*Cohen*_ *=* *0,112*	*d*_*Cohen*_ *=* *0,061*	*d*_*Cohen*_ *=* *0,016*	*d*_*Cohen*_ *=* *−0,018*	*d*_*Cohen*_ *=* *−0,178*	*d*_*Cohen*_ *=* *0,073*
Ja	82 (6,9 %)	6,01 (1,76)*	5,54 (1,55)	5,44 (1,72)	1,60 (1,25)	1,54 (1,22)	2,20 (1,68)	1,84 (1,30)
Nein	1108 (93,1 %)	6,40 (1,47)*	5,71 (1,51)	5,54 (1,62)	1,62 (1,27)	1,52 (1,19)	1,93 (1,50)	1,95 (1,51)
*Chronische Erkrankung (N* *=* *1677)*	–	*d*_*Cohen*_ *=* *0,000*	*d*_*Cohen*_ *=* *0,070*	*d*_*Cohen*_ *=* *0,071*	*d*_*Cohen*_ *=* *−0,029*	*d*_*Cohen*_ *=* *−0,095*	*d*_*Cohen*_ *=* *−0,019*	*d*_*Cohen*_ *=* *−0,129*
Ja	539 (32,1 %)	6,29 (1,59)	5,53 (1,59)	5,36 (1,71)	1,71 (1,46)	1,65 (1,41)	2,07 (1,67)	2,13 (1,69)*
Nein	1138 (67,9 %)	6,29 (1,61)	5,64 (1,57)	5,48 (1,68)	1,67 (1,32)	1,53 (1,19)	2,04 (1,58)	1,93 (1,48)*

„Als gefährlicher dargestellt“ = „Das Virus wird absichtlich als gefährlich dargestellt, um die Öffentlichkeit in die Irre zu führen“, „Expert*innen täuschen“ = „Expert*innen täuschen uns absichtlich und zu ihrem eigenen Vorteil, obwohl das Virus eigentlich nicht schlimmer ist als eine Grippe“, „Expert*innen glauben“ = „Man sollte den Expert*innen glauben, wenn sie sagen, dass das Virus gefährlich ist“ (invertiert), „Corona-Profit“ = „Die Corona-Krise wurde so groß geredet, damit einige wenige von ihr profitieren“

*d*_*Cohen*_ (Cohens d, 1988) und η^2^ (Eta-Quadrat) zur Ermittlung der Effektstärke

^a^Im Vergleich zu 51- bis 60-Jährigen

^b^Im Vergleich zu 61- bis 70-Jährigen

^c^Im Vergleich zum Pflegepersonal

^d^Im Vergleich zu medizinisch-technischem Personal

^e^Im Vergleich zu Verwaltungspersonal

^f^Im Vergleich zu wissenschaftlichem Personal

Signifikanz des Unterschieds zwischen den Gruppen, ermittelt durch t‑Test und ANOVA (*analysis of variance* bzw. Varianzanalyse): *** *p* < 0,001, ** *p* < 0,01, * *p* < 0,05

### Einstellung zu Impfungen, Verschwörungsannahmen und Kommunikation unter den einzelnen Berufsgruppen

Die Impfbereitschaft war unter Ärzt*innen mit einem Mittelwert von 6,81 (SD = 0,78) am höchsten und unterschied sich signifikant von der Impfbereitschaft unter Pflegekräften, medizinisch-technischem Personal und Verwaltungspersonal (*p* < 0,001, η^2^ = 0,005). Auch zwischen den Geschlechtern war ein signifikanter Unterschied zu verzeichnen, jedoch ohne großen Effekt (Frauen: M = 6,22, SD = 1,67; Männer: M = 6,55, SD = 1,23; *p* < 0,001, d = 0,208). Jüngere waren tendenziell weniger impfbereit, besonders im Vergleich zu den ältesten Befragten (M = 6,06, SD = 1,77; *p* < 0,05; η^2^ = 0,009). Befragte, die sich bereits mit SARS-CoV‑2 infiziert hatten, waren signifikant weniger impfbereit als diejenigen, die sich noch nicht infiziert hatten (Infektion: M = 6,01, SD = 1,76; keine Infektion: M = 6,40, SD = 1,47; *p* < 0,05, d = 0,261). Bezüglich der empfundenen Sicherheit der Impfung gab es zwar ebenfalls einen Geschlechtsunterschied (Frauen: M = 5,53, SD = 1,62; Männer: M = 5,86, SD = 1,37; *p* < 0,001, d = 0,210), jedoch keinerlei signifikante Unterschiede bezüglich des Alters oder der Berufsgruppe. Signifikante Unterschiede traten hinsichtlich der empfundenen Effektivität der Impfung auf: Ärzt*innen schätzten die Effektivität der Impfung signifikant höher ein als die anderen Berufsgruppen, mit Ausnahme vom wissenschaftlichen Personal (M = 5,99, SD = 1,22; *p* < 0,001; η^2^ = 0,002). Befragte, die Kontakt zu COVID-19-Patient*innen hatten, beurteilten die Effektivität der Impfung besser als diejenigen, die keinen Kontakt hatten (Kontakt: M = 5,53, SD = 1,69; kein Kontakt: M = 5,32, SD = 1,73; *p* < 0,05; d = −0,123).

Ärzt*innen und das wissenschaftliche Personal stimmten allen Verschwörungsaussagen signifikant weniger zu als die anderen Berufsgruppen. Die höchste Zustimmung der Ärzt*innen und des wissenschaftlichen Personals findet sich für die invertierte Aussage: „Man sollte Expert*innen glauben, wenn sie sagen, dass das Virus gefährlich ist“ (Ärzt*innen: M = 1,72, SD = 1,38; wissenschaftliches Personal: M = 1,69, SD = 1,22; η^2^ = 0,001). Die größte Zustimmung erhielt die Aussage: „Die Corona-Krise wurde so groß geredet, damit einige wenige von ihr profitieren“, welche zudem die größte Effektstärke unter den Verschwörungsaussagen aufwies (η^2^ = 0,008). Auch dieser Aussage stimmten Ärzt*innen und das wissenschaftliche Personal am wenigsten, das Verwaltungspersonal jedoch am meisten zu (Ärzt*innen: M = 1,40, SD = 0,94; wissenschaftliches Personal: M = 1,60, SD = 1,18; Verwaltungspersonal: M = 2,35, SD = 1,80; Signifikanz des Unterschiedes zwischen Ärzt*innen/wissenschaftlichem und Verwaltungspersonal: *p* < 0,001).

Die Kommunikation in der Klinik wurde durchweg als eher gut eingeschätzt, lediglich die Aussage zur Möglichkeit des Einbringens gewann weniger Zustimmung (M = 3,26, SD = 2,01, Tab. 2). Signifikante Unterschiede zwischen den Berufsgruppen finden sich nur bezüglich der Transparenz der Entscheidungsabläufe und des Informationsflusses von Führungskräften/Vorstand zu den Mitarbeitenden. Die Entscheidungsabläufe wurden vom wissenschaftlichen Personal am transparentesten eingeschätzt (M = 5,18, SD = 1,63), sie unterschieden sich signifikant von den Ärzt*innen und den Pflegekräften, welche die geringsten Zustimmungswerte aufwiesen (Ärzt*innen: M = 4,62, SD = 1,99; Pflegekräfte: M = 4,67, SD = 1,84; *p* < 0,05; η^2^ = 0,013). Den Informationsfluss von den Führungskräften zu den Mitarbeitenden schätzten die Pflegekräfte am besten (M = 4,82, SD = 1,79), das Verwaltungspersonal am schlechtesten ein (M = 4,46, SD = 1,86; *p* < 0,05; η^2^ = 0,003). Das Verwaltungspersonal hingegen schätzte den Informationsfluss vom Vorstand zu den Mitarbeitenden am besten ein (M = 4,88, SD = 1,74), die Pflegekräfte am schlechtesten (M = 4,46, SD = 1,95; *p* < 0,05; η^2^ = 0,007). Die Effektstärken aller Kommunikations-Items waren gering (η^2^ < 0,06).

**Table 2:** 

	Ausreichende Informiertheit über Maßnahmen M (SD)	Transparente Entscheidungsabläufe M (SD)	Rechtzeitiges informieren über Entscheidungen M (SD)	Möglichkeit des Einbringens eigener Erfahrungen und Ideen M (SD)	Guter Informationsfluss von Führungskräften zu Mitarbeitenden M (SD)	Guter Informationsfluss von Vorstand zu Mitarbeitenden M (SD)
**Gesamtstichprobe (*N*** **=** **1683)**	5,20 (1,41)	4,87 (1,76)	4,94 (1,71)	3,26 (2,01)	4,63 (1,85)	4,62 (1,86)
	η^2^ = 0,001	η^2^ = 0,013	η^2^ = 0,003	η^2^ = 0,001	η^2^ = 0,003	η^2^ = 0,007
**Berufsgruppen**						
Ärzt*innen	5,14 (1,54)	4,62 (1,99)*^a,b^	4,68 (2,00)	3,55 (1,93)	4,62 (2,01)	4,46 (2,03)
Pflegepersonal	5,20 (1,49)	4,67 (1,84)*^a,b^	4,90 (1,82)	3,26 (2,05)	4,82 (1,79)*^a^	4,46 (1,95)*^a^
Medizinisch-technisches Personal	5,17 (1,29)	4,98 (1,64)	5,01 (1,56)	3,12 (1,98)	4,58 (1,76)	4,60 (1,73)
Verwaltung	5,16 (1,34)	5,06 (1,64)	5,05 (1,56)	3,25 (2,02)	4,46 (1,86)	4,88 (1,74)
Wissenschaftliches Personal	5,38 (1,33)	5,18 (1,63)	5,01 (1,56)	3,21 (1,99)	4,55 (1,90)	4,78 (1,79)

η^2^ (Eta-Quadrat) zur Ermittlung der Effektstärke

^a^Signifikanter Mittelwertunterschied gegenüber Verwaltung

^b^Signifikanter Mittelwertunterschied gegenüber dem wissenschaftlichen Personal

Signifikanz des Unterschieds zwischen den Gruppen, ermittelt durch t‑Test und ANOVA: *** *p* < 0,001, ** *p* < 0,01, * *p* < 0,05

### Zusammenhang von Impfbereitschaft, Verschwörungsannahmen sowie Vertrauen in Information und Kommunikation

Die Ergebnisse zu den Aussagen zur Kommunikation werden in Tab. 3 dargestellt. Impfbereitschaft war signifikant mit der empfundenen Sicherheit und Effektivität der Impfung korreliert (Sicherheit: rho = 0,613; Effektivität: rho = 0,566; *p* < 0,001). Die Kommunikations-Items hatten, ausgenommen der ausreichenden Informiertheit, eine sehr schwache, jedoch signifikante positive Assoziation mit Impfbereitschaft. Ähnliche Ergebnisse zeigten sich für die empfundene Effektivität und Sicherheit. Alle Verschwörungs-Items wiesen eine mittelgradige negative Assoziation mit der Impfbereitschaft auf. Der größte Zusammenhang mit der Impfbereitschaft war zu beobachten für die empfundene Möglichkeit, sich einbringen zu können (Sicherheit: rho = 0,114; Effektivität: rho = 0,120; *p* < 0,001). Die invertierte Aussage: „Man sollte Expert*innen glauben, wenn sie sagen, dass das Virus gefährlich ist“, wies die größte Assoziation zur Impfbereitschaft auf. Auch mit der empfundenen Sicherheit und Effektivität sowie den Kommunikations-Items sind die Annahmen signifikant negativ korreliert. Bedeutsam erscheint hier vor allem das Gefühl, sich einbringen zu können: Dieses wies zwar wie die anderen Items nur eine sehr schwache Assoziation auf, jedoch die größte unter den Kommunikations-Items.

**Table 3:** 

	Impfbereitschaft	Sicherheit	Effektivität	Als gefährlicher dargestellt	Expert*innen täuschen	Expert*innen glauben	Corona-Profit
*Impfbereitschaft*	1	–	–	−0,385***	−0,393***	−0,443***	−0,417***
*Glaube an*							
Sicherheit COVID-19-Impfung	0,613***	1	–	−0,395***	−0,416***	−0,448***	−0,431***
Effektivität COVID-19-Impfung	0,566***	0,566***	1	−0,411***	−0,433***	−0,428***	−0,414***
*Einschätzung der Kommunikation*							
Ausreichende Informiertheit über Maßnahmen	0,035	0,019	0,024	−0,036	−0,053*	−0,028	−0,012
Transparente Entscheidungsabläufe	0,092***	0,076**	0,071**	−0,033	−0,034	−0,070**	−0,059*
Rechtzeitiges Informieren über Entscheidungen	0,076**	0,075**	0,069**	−0,014	−0,038	−0,053*	−0,048*
Möglichkeit des Einbringens eigener Erfahrungen und Ideen	0,103***	0,114***	0,120***	−0,098***	−0,093***	−0,120***	−0,097***
Guter Informationsfluss von Führungskräften zu Mitarbeitenden	0,084***	0,081***	0,071**	−0,054*	−0,056*	−0,069**	−0,070**
Guter Informationsfluss von Vorstand zu Mitarbeitenden	0,085***	0,083***	0,077**	−0,046	−0,053*	−0,093***	−0,056*

„Als gefährlicher dargestellt“ = „Das Virus wird absichtlich als gefährlich dargestellt, um die Öffentlichkeit in die Irre zu führen“, „Expert*innen täuschen“ = „Expert*innen täuschen uns absichtlich und zu ihrem eigenen Vorteil, obwohl das Virus eigentlich nicht schlimmer ist als eine Grippe“, „Expert*innen glauben“ = „Man sollte den Expert*innen glauben, wenn sie sagen, dass das Virus gefährlich ist“ (invertiert), „Corona-Profit“ = „Die Corona-Krise wurde so groß geredet, damit einige wenige von ihr profitieren“

*** *p* < 0,001, ** *p* < 0,01, * *p* < 0,05

### Prädiktoren für Impfbereitschaft

Im nächsten Schritt wurde eine Regressionsanalyse mit der Impfbereitschaft als Outcome-Variable berechnet, die Ergebnisse sind in Tab. 4 dargestellt. Die erklärte Varianz des Modells beträgt R^2^_adjusted_ = 0,562 (*p* < 0,001). Die stärkste Assoziation haben die Einstellungen zu den Impfstoffen, wobei jedoch nur die empfundene Sicherheit eine signifikante Assoziation aufweist (B = 0,431; b = 0,483; Standardfehler = 0,037; *p* < 0,001). Schwache Zusammenhänge sind für das Alter zu finden: Verglichen mit den mittleren Altersgruppen berichteten die 31- bis 40-Jährigen und die beiden ältesten Altersgruppen ab 51 Jahren eine höhere Impfbereitschaft. Der letzte Faktor, der eine signifikante Assoziation mit der Impfbereitschaft hatte, ist der Verschwörungsglaube. Hier zeigt sich ein negativer Zusammenhang (B = −0,301; b = −0,235; Standardfehler = 0,036; *p* < 0,001). Geschlecht, Berufsgruppe, Kommunikation, Zeitpunkt der Befragung sowie Kontakt zu COVID-19-Patient*innen, vergangene SARS-CoV-2-Infektion und chronische Erkrankung der befragten Person wiesen keine signifikanten Assoziationen auf.

**Table 4:** 

Unabhängige Variablen	R^2^ = 0,571; R_adjusted_ = 0,562; F(18,847) = 62,701, *p* < 0,001					
	Unstandardisierte Koeffizienten	Std.-Fehler	Standardisierte Koeffizienten	*p*	95 % KI: unterer Wert	95 % KI: oberer Wert
*Konstante*	3,554	0,265	–	**0,000**	2,771	4,336
*Geschlecht (männlich* *=* *Ref.)*						
Weiblich	−0,078	0,110	−0,022	0,451	−0,280	0,125
*Altersgruppe (Jahre; 18–30* *=* *Ref.)*						
31–40	0,293	0,105	0,089	**0,008**	0,077	0,509
41–50	0,129	0,109	0,037	0,259	−0,095	0,354
51–60	0,327	0,109	0,097	**0,005**	0,101	0,553
61–70	0,428	0,170	0,065	**0,010**	0,101	0,755
Berufsgruppe (Ärzt*innen *=* *Ref.)*						
Pflegepersonal	−0,093	0,092	−0,031	0,261	−0,256	0,069
Medizinisch-technisches Personal	0,030	0,113	0,008	0,794	−0,194	0,253
Verwaltung	0,020	0,143	0,004	0,910	−0,325	0,365
Wissenschaftliches Personal	0,073	0,191	0,010	0,708	−0,311	0,458
Kontakt zu COVID-19-Patient*innen (Ja)	0,023	0,122	0,007	0,791	−0,147	0,193
Positiver COVID-19-Test (Ja)	−0,201	0,074	−0,037	0,080	−0,426	0,024
Chronische Erkrankung (Ja)	0,072	0,037	0,022	0,356	−0,081	0,225
*Einstellungen zur Impfung*						
Sicherheit COVID-19-Impfung	0,431	0,037	0,483	**0,000**	0,313	0,549
Effektivität COVID-19-Impfung	0,112	0,040	0,117	0,069	−0,009	0,233
Verschwörung	−0,301	0,036	−0,235	**0,000**	−0,406	−0,195
Kommunikation	0,039	0,026	0,034	0,185	−0,018	0,096
Zeitvariable (nach Aussetzen)	−0,108	0,140	−0,036	0,382	−0,350	0,134
Zeitvariable*Geschlecht	0,165	0,160	0,047	0,273	−0,131	0,461

Stichprobe *N* = 1683. Abhängige Variable ist die Impfbereitschaft, codiert von 1–7. Die Zeitvariable ist codiert in 0 = vor Aussetzung des Impfens mit Vektorimpfstoffen für Personen unter 60, 1 = nach Aussetzung

*R*^*2*^_*adjusted*_ adjustiertes R^2^ des Modells, *P‑Wert* Signifikanz des Modells bzw. des Koeffizienten, *KI* Konfidenzintervall

## Diskussion

Die Impfbereitschaft des Krankenhauspersonals war in dieser Studie im Vergleich zur Gesamtbevölkerung sehr hoch [31]. Sie variierte zwischen den Berufsgruppen und war unter den Ärzt*innen mit M = 6,81 (SD = 0,78) am höchsten und unter dem Verwaltungspersonal am geringsten (M = 6,05, SD = 1,85), was Ergebnisse aus früheren Studien bestätigt [12]. Dies könnte damit zusammenhängen, dass Personen mit einem höheren Bildungsabschluss die Effizienz und die Sicherheit der Impfung höher einschätzen, was wiederum positiv mit einer hohen Impfbereitschaft assoziiert ist [32]. Insgesamt stimmte das Krankenhauspersonal den Verschwörungsannahmen wenig zu. Unter den Berufsgruppen stimmte das Verwaltungspersonal den Aussagen tendenziell am ehesten zu, während Ärzt*innen und das wissenschaftliche Personal die kleinsten Zustimmungswerte aufwiesen. Ergebnisse früherer Arbeiten lassen vermuten, dass der forschungsnahe Hintergrund der Ärzt*innen und des wissenschaftlichen Personals dazu führt, dass diese wissenschaftlichen Erkenntnissen eher Vertrauen schenken bzw. weniger unter Kontrollverlust leiden [22, 33, 34]. Bestätigt wurde dies während der Befragung dadurch, dass Ärzt*innen der invertierten Aussage, in der es darum geht, den Expert*innen zu glauben, wenn sie sagen, dass das Virus gefährlich ist, am ehesten zustimmten. Dies könnte daran liegen, dass Ärzt*innen sich selbst als Expert*innengruppen angesprochen fühlen und selbstverständlich ihrer eigenen Expertise vertrauen.

In der Regressionsanalyse der Gesamtstichprobe unter Berücksichtigung der Einflussgrößen Verschwörungsglaube, Geschlecht, Alter, Berufsgruppen, empfundene Sicherheit und Effektivität der Impfung, Kommunikation und Vertrauen in die Informationen innerhalb der Klinik sowie Zeitpunkt der Befragung konnte eine signifikante positive Assoziation des Alters auf die Impfbereitschaft beobachtet werden. So waren ältere Personen tendenziell eher dazu bereit, sich impfen zu lassen. Jedoch zeigte sich entgegen vorhergegangener Forschungsergebnisse, dass weder Geschlecht noch Berufsgruppe eine signifikante Assoziation mit Impfbereitschaft aufwies, was die erste Hypothese dieser Arbeit zum Teil widerlegt [2, 7, 11].

Die positiven Einschätzungen der Wirksamkeit und Sicherheit der Impfung wiesen eine signifikante positive Assoziation mit Impfbereitschaft auf. Dies unterstreicht die in der Theorie der Schutzmotivation betonte Rolle der Selbstwirksamkeit- und Handlungsergebniserwartung (s. oben; [5]) und spiegelt die Ergebnisse vorangegangener Studien wider, in denen vor allem die Rolle der Aktionskosten unterstrichen wurde [2, 5, 14–17]. Der zweite wichtige Aspekt, die Bedrohungseinschätzung, wurde in der vorliegenden Studie mittels der Verschwörungstheorien gemessen. Hier zeigte sich in den vorangegangenen Deskriptivanalysen, dass vor allem die invertierte Aussage zum Glauben an die Einschätzungen der Expert*innen und die Aussage, dass aus der Pandemie von einigen wenigen Profit gezogen würde, negativ mit der Impfbereitschaft assoziiert sind. Erwiesenermaßen sind Personen besonders anfällig für Verschwörungsannahmen, wenn das individuelle Risiko einer COVID-19-Erkrankung zwar als gering, dafür aber das Risiko sozialer und seelischer Einbußen durch die politischen Maßnahmen zur Eindämmung der Pandemie als groß angesehen wird [35]. Basierend auf den Ergebnissen anderer Studien [1, 24, 36] könnte die Politik mehr auf Fehlinformationen in sozialen Medien eingehen und die Diskussion um Impfungen mitgestalten, damit zugleich das Vertrauen in die Maßnahmen staatlicher Institutionen aufrechterhalten wird.

Hilfreich könnten hierbei Erkenntnisse aus der Inokulationstheorie sein. Diese Theorie bedient sich einer Analogie zur medizinischen Inokulation, also der artifiziell induzierten Immunisierung, und erklärt dabei, wie bestehende Einstellungen von Individuen gegenüber Veränderungsversuchen resistent gemacht werden können [38]. Ein besonderer Fokus wird hier auf Desinformationen unter anderem durch Medien, Werbung und Gruppendruck gelegt. Für den Aufbau einer Resistenz gegenüber Desinformationen werden nach der Inokulationstheorie folgende Mittel verwendet: Zunächst soll dem Individuum bewusstgemacht werden, dass es vulnerabel ist gegenüber sogenannten *persuasion attacks*, sprich: Versuchen, das Individuum von seinen eigenen Einstellungen abzubringen und von anderen zu überzeugen. Danach wird das Individuum schwachen Gegenargumenten zu dessen eigenen Einstellungen ausgesetzt, um diese dann durch bereitgestellte Argumente für die Einstellung des Individuums zu widerlegen [39]. Damit wird verhindert, dass sich der Verschwörungsglaube verfestigt, und Skepsis gegenüber Verschwörungsaussagen gefördert [38]. Erste Studien belegten, dass dieses Verfahren die Anfälligkeit für Verschwörungsaussagen reduziert [38]. In Bezug auf die COVID-19-Pandemie wäre denkbar, dass die Bevölkerung gegenüber der Verbreitung von Verschwörungsannahmen mehr sensibilisiert wird und lernt, fehlerhafte Argumentation zu identifizieren [40]. Dies könnte unter anderem in der Schule oder am Arbeitsplatz im Rahmen eines Workshops zu Desinformationen oder einer politischen Aufklärungskampagne geschehen.

Eine gute Kommunikation innerhalb der Kliniken und der Zeitpunkt der Befragung (vor und nach dem Aussetzen der Impfungen mit dem AstraZeneca-Impfstoff) hatten in der Regressionsanalyse keinen signifikanten Einfluss auf die Impfbereitschaft. Frühere Studien zu Grippeimpfungen zeigten aber, dass die Gefahrenkommunikation unter zögerlichen Menschen auch einen negativen Einfluss auf die Impfbereitschaft haben kann [37]. Dies sollte auch in Bezug auf COVID-19 näher untersucht werden.

## Limitationen

Trotz der großen Stichprobe ist zu beachten, dass es sich bei der vorliegenden Studie um eine anonyme Onlinebefragung handelte und nicht auszuschließen ist, dass eher Personen mit Vertrauen in die Wissenschaft an der Studie teilnahmen und somit ein *Self-Selection Bias* bestehen könnte. Es handelte sich zudem um eine Querschnittsbefragung, weshalb ein Rückschluss auf die Kausalität der Prädiktoren auf die Impfbereitschaft nicht gegeben ist. Für das Zustandekommen einer Impfung dürften außerdem noch zahlreiche, nicht erfasste Faktoren eine Rolle spielen, wie beispielsweise der zeitliche und organisatorische Aufwand der Impfung. Zudem wurden lediglich die empfundene Wirksamkeit und die Sicherheit der Impfung erfasst; weitere wesentliche Aspekte der Theorie der Schutzmotivation wurden nicht erhoben. Es wurde außerdem nicht erfasst, ob die Befragten zum Zeitpunkt der Befragung bereits geimpft waren.

Das Thema Verschwörungsannahmen wurde zwar in der Öffentlichkeit diskutiert, jedoch fehlen bislang standardisierte Verfahren für die Erfassung des Glaubens an Verschwörungsannahmen. Wir nutzten Items zweier bewährter Skalen aus der COSMO-Studie [27] und einer Repräsentativbefragung [1], die stark zusammenhängen. Ein weiterer nicht erfasster Faktor im Bereich der Verschwörungsannahmen und der Impfbereitschaft könnten extreme politische Gesinnungen sein, die zu Verschwörungsannahmen prädisponieren [3, 23].

## Footnotes

1 Da Vaxzevria in der Bevölkerung vor allem unter dem Namen „AstraZeneca“ bekannt ist, wurde dieser im Folgenden weiterverwendet.
